# Lecithin:cholesterol acyltransferase: symposium on 50 years of biomedical research from its discovery to latest findings

**DOI:** 10.1194/jlr.S120000720

**Published:** 2020-06-01

**Authors:** Kaare R. Norum, Alan T. Remaley, Helena E. Miettinen, Erik H. Strøm, Bruno E. P. Balbo, Carlos A. T .L. Sampaio, Ingrid Wiig, Jan Albert Kuivenhoven, Laura Calabresi, John J. Tesmer, Mingyue Zhou, Dominic S. Ng, Bjørn Skeie, Sotirios K. Karathanasis, Kelly A. Manthei, Kjetil Retterstøl

**Affiliations:** * Department of Nutrition, University of Oslo, Oslo, Norway; † National Institutes of Health, Bethesda, MD; § Department of Medicine, University of Helsinki and University Central Hospital, Helsinki, Finland; ‡ Departments of Pathology Oslo University Hospital, Oslo, Norway; ‖‖ Anesthesiology, Oslo University Hospital, Oslo, Norway; # Centre for Rare Disorders, Oslo University Hospital, Oslo, Norway; *** Department of Endocrinology, Morbid Obesity, and Preventive Medicine, Lipid Clinic, Oslo University Hospital, Oslo, Norway; ‖ Division of Nephrology and Molecular Medicine Department of Medicine, University of São Paulo School of Medicine, São Paulo, Brazil; $ Department of Pediatrics, Section Molecular Genetics, University Medical Center Groningen, University of Groningen, Groningen, The Netherlands; ** Center E. Grossi Paoletti, Department of Pharmacological and Biomolecular Sciences, Università degli Studi di Milano, Milan, Italy; †† Department of Biological Sciences, Purdue University, West Lafayette, IN; §§ Cardiometabolic Disorder Research, AMGEN, San Francisco, CA; ‡‡ Department of Medicine, University of Toronto and Keenan Research Center, Li Ka Shing Knowledge Institute, St. Michael’s Hospital, Toronto, Canada; ## NeoProgen, Baltimore, MD; $$ Life Sciences Institute, University of Michigan, Ann Arbor, MI

**Keywords:** HDL cholesterol, lipoprotein, cardiovascular heart disease, lipoprotein X

## Abstract

LCAT converts free cholesterol to cholesteryl esters in the process of reverse cholesterol transport. Familial LCAT deficiency (FLD) is a genetic disease that was first described by Kaare R. Norum and Egil Gjone in 1967. This report is a summary from a 2017 symposium where Dr. Norum recounted the history of FLD and leading experts on LCAT shared their results. The Tesmer laboratory shared structural findings on LCAT and the close homolog, lysosomal phospholipase A2. Results from studies of FLD patients in Finland, Brazil, Norway, and Italy were presented, as well as the status of a patient registry. Drs. Kuivenhoven and Calabresi presented data from carriers of genetic mutations suggesting that FLD does not necessarily accelerate atherosclerosis. Dr. Ng shared that LCAT-null mice were protected from diet-induced obesity, insulin resistance, and nonalcoholic fatty liver disease. Dr. Zhou presented multiple innovations for increasing LCAT activity for therapeutic purposes, whereas Dr. Remaley showed results from treatment of an FLD patient with recombinant human LCAT (rhLCAT). Dr. Karathanasis showed that rhLCAT infusion in mice stimulates cholesterol efflux and suggested that it could also enhance cholesterol efflux from macrophages. While the role of LCAT in atherosclerosis remains elusive, the consensus is that a continued study of both the enzyme and disease will lead toward better treatments for patients with heart disease and FLD.

## IN MEMORIAM: KAARE R. NORUM (1932–2019)

**Fig. 1.**
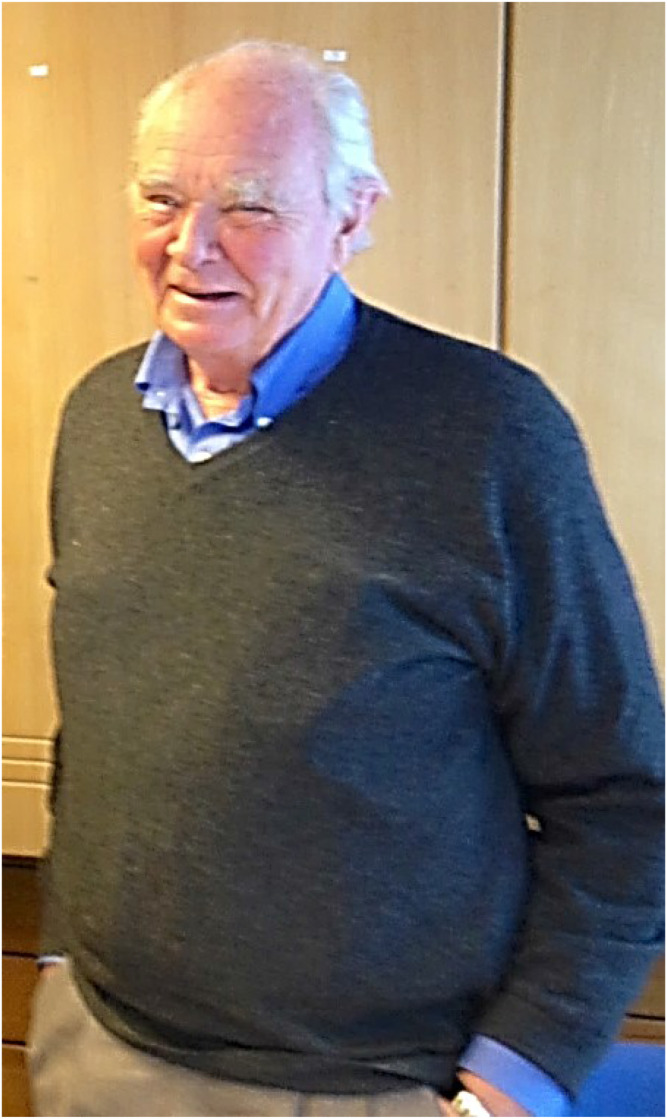
Kaare R. Norum. Photo taken in 2016 by K. Retterstøl.

Dr. Kaare R. Norum, our dear colleague and professor at the University of Olso, passed away on November 22, 2019 while in the process of preparing this article (**Fig. 1**). He was the first to describe familial LCAT deficiency (FLD), a rare disease associated with the absence or defective function of LCAT and low HDL plasma levels. This report is a summary of the proceedings from a symposium Dr. Norum organized in Oslo, Norway, on May 19, 2017, and it is dedicated to him. Dr. Norum was an astute clinical observer and a dedicated scientist committed to the understanding of fundamental scientific processes underpinning clinical observations and their translation back to the clinic with the ultimate aim to help patients. Dr. Norum worked tirelessly to build an international network of clinical and basic scientists to both advance the science and help patients around the world. Dr. Norum had a big heart and was fond of a good joke. We will miss you, Dr. Norum.

In May 2017, a symposium entitled “LCAT-deficiency: from phenotype to genotype and beyond” was held at the University of Oslo to mark the fiftieth anniversary of the first report on FLD. Leading experts with a broad range of experience, from clinical to basic research, shared their latest knowledge of LCAT. This report aims to summarize the data presented.

## THE DISCOVERIES OF LCAT AND FLD: TWO SERENDIPITIES (K. R. Norum)

In 1962, John A. Glomset et al. (1) at the University of Washington published experiments suggesting the existence of a transferase capable of catalyzing the transesterification of fatty acids between the hydroxyl groups of cholesterol and of the glycerol of both neutral and acidic lipids. This led to the discovery of the enzyme LCAT (2).

In 1966, Kaare R. Norum was developing a method for the evaluation of different types of hyperlipidemias. By chance, he found an unusual electrophoretic profile in the blood plasma of one patient. There was no HDL or preβ-lipoprotein in the plasma of the patient, but it contained high amounts of both triglycerides and cholesterol. Almost all the plasma cholesterol was unesterified, whereas about 80–90% of the total serum cholesterol is esterified in normal plasma. He also found that there was no LCAT activity in the serum. The patient had an opaque cornea and related that her two sisters had the same symptoms, and they were also referred to the University Hospital. Egil Gjone, the doctor in charge of the patients, and Norum began a collaboration to further study this family. None of these patients had LCAT activity in their serum, and a new inborn error of metabolism, FLD, had been discovered and was subsequently published in 1967 (3).

When Glomset learned that Norum and Gjone had described a disease caused by the lack of LCAT, he eagerly contacted them. This led to long-lasting and very fruitful research collaboration between Oslo and Seattle. These investigators showed that the lipoproteins in the plasma of the FLD patients were very different from those of healthy patients, and in particular, the patients’ plasma lacked HDL. In addition, there were large amounts of membranous particles in the plasma from these patients. The membranes consisted mainly of free cholesterol and phospholipids organized into multilamellar vesicles, now referred to as lipoprotein X (LpX) (4).

**Fig. 2.**
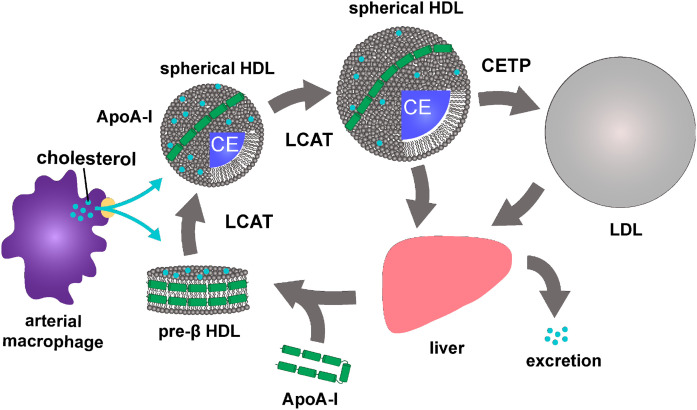
Reverse cholesterol transport. Cholesterol is effluxed from arterial macrophages to preβ-HDL, where it is esterified by LCAT. The cholesteryl ester (CE) then enters the center of the HDL and promotes maturation to larger and more spherical particles. HDL transports CEs directly to the liver, or via LDL when they are transferred by CETP. Cholesterol is then excreted in bile and other components are recycled.

Further study of the lipoproteins in the blood of the FLD patients led to a better understanding of the normal transport of cholesterol in the body (**Fig. 2**). LCAT, which binds to HDL particles, converts chylomicron surface lipids to cholesteryl esters and lysolecithin. In addition, cholesterol and lecithin in liver-derived VLDLs, which are secreted by the liver, are transferred to HDL where the cholesterol is esterified by LCAT. HDL is mainly produced in the liver, and it is delivered to the blood plasma as nascent small ellipsoid HDL particles, consisting mainly of a protein, called apoA-I produced in liver and intestine and small amounts of cholesterol. The free cholesterol in nascent HDL particles is esterified by LCAT in blood, and because of its hydrophobic nature, esterified cholesterol moves into the core of the HDL particle. HDL can also take up free cholesterol from cells in a process referred to as cellular cholesterol efflux. The surplus of esterified cholesterol in HDL is subsequently transferred to LDL by the plasma protein cholesterol ester transfer protein (CETP), and eventually delivered to the liver LDL-receptors. Surplus esterified cholesterol in HDL is also transferred to the liver directly via SRBI receptors present in the liver, but this process is of less quantitative significance in humans (5). This entire process whereby cellular cholesterol is effluxed to HDL, esterified by LCAT, transferred to LDL by CETP, and delivered to the liver by LDL is referred to as reverse cholesterol transport, a terminology first introduced by Glomset in 1968 (6) (Fig. 2). The key role that LCAT plays in reverse cholesterol transport is in agreement with the animal data detailed below.

In healthy patients, the breakdown of chylomicrons, the conversion of VLDL to LDL, and the transformation of nascent HDL are rapid processes that are thus difficult to examine. However, in patients with FLD, these lipoprotein conversions are blocked. In experiments involving incubations of FLD patient plasma with purified LCAT, Norum, Gjone, and Glomset were able to observe and study these processes delineating important steps in lipoprotein conversion and mature normal lipoprotein biogenesis in plasma.

The main symptoms of FLD patients are renal failure and corneal opacities. LpX accumulation in tissue appears to be the main cause of kidney injury (4, 7). Similar LpX accumulation in the cornea may also contribute to corneal opacities and impaired vision in these patients. However, LpX does not seem to be the main factor leading to impaired vision in other lipoprotein disorders, such as partial deficiency of LCAT, referred to as fish-eye disease (FED), and apoA-I deficiency. FED patients, like FLD patients, have low HDL-cholesterol but milder symptoms of corneal opacities. Clearly, more work is needed to clarify whether LpX, HDL deficiency, or both contribute to the development of corneal opacities and visual impairments in these patients. A review containing the signs and symptoms, clinical data, and results of the research with these patients has been published as a chapter in *The Metabolic Basis of Inherited Disease* (8).

There has been, and still is, a great interest in LCAT. According to PubMed as of January 2020, there were more than 2,000 published papers on LCAT. The complete or partial lack of LCAT is a very rare genetic disease with only about 100 patients that are known globally. FLD is a devastating disease with no good available therapies. Studies on the molecular pathophysiology of FLD not only provide hope for a future treatment for these patients, but also provide valuable information on cholesterol metabolism, transport, and function. Thus, their study could unveil novel insights for new therapeutic targets of disorders in lipoprotein metabolism and cardiovascular disease beyond FLD.

## STRUCTURE AND FUNCTION OF LCAT (J. J .G. Tesmer and K. A. Manthei)

**Fig. 3.**
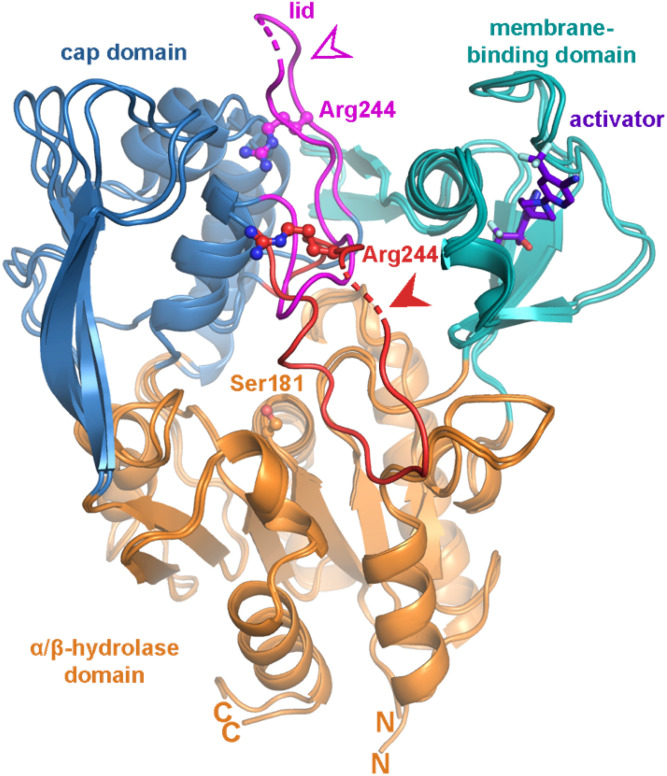
Crystal structures reveal a dynamic lid that shields the active site of LCAT. Two crystal structures are overlaid to highlight lid movement. The closed conformation (Protein Data Bank code 5TXF) is indicated with a filled arrowhead and red lid, and an open state of LCAT (Protein Data Bank code 6MVD) bound to an activator (purple) is indicated with an open arrowhead and magenta lid. Unmodeled residues in the lid are indicated by a dashed line. The α/β-hydrolase domain is colored in orange, the cap domain in blue, and the membrane binding domain in teal. The active site residue Ser181 is indicated with orange ball-and-sticks, whereas Arg244, which is mutated in FLD, is shown in the lid with red and magenta ball-and-sticks. N and C indicate the amino and carboxyl termini, respectively, observed in the structures.

The closest homolog of LCAT in the human genome is lysosomal phospholipase A2 (LPLA2), and together they constitute a family of phospholipases that transfer the acyl chain from lipids, such as lecithin, to acceptor lipids, such as ceramide or cholesterol. LPLA2 and LCAT are important for lung surfactant catabolism and reverse cholesterol transport to the liver via HDL, respectively, and are both important targets for biotherapeutic development. The Tesmer laboratory has determined atomic resolution crystal structures for both LPLA2 and LCAT (9–11) and is working toward understanding the molecular mechanisms underlying disease-causing variants as well as small molecular activators that could be used to treat acute coronary syndrome and/or fatal genetic diseases such as FLD. These structure-based studies have allowed for the creation of an accurate map of the position of disease-causing mutations and have revealed unexpected structural features that are believed to be involved in membrane and substrate binding and in catalysis. A notable structural difference between LPLA2 and LCAT is found in a flexible lid element that blocks access to the active site of LCAT. The molecular basis for LCAT activation by apoA-I-containing HDLs is therefore hypothesized to involve retraction of this lid element in a form of interfacial activation (10). Based on multiple crystal structures and hydrogen deuterium exchange data, it is now apparent that this dynamic lid is able to protect the LCAT active site from solvent (**Fig. 3**) (10).

The structure of LCAT bound to an LCAT-activating compound as well as an acyl intermediate state mimic, isopropyl dodecylfluorophosphonate, has also been reported (Fig. 3) (11). The combination of these two ligands promotes a lid-open conformation and full access to the active site. The Tesmer laboratory has confirmed that the activator compound binds to an allosteric site via site-directed mutants that both eliminate compound binding and decrease the thermal stability of LCAT. The activator enhances the acyltransferase activity of LCAT R244H in vitro. Such studies pave the way for the design of rational drugs or improved biotherapeutics that may be used to treat coronary heart disease (CHD) and the renal disease that results from FLD. The new structure also allows for a better understanding of the acyl binding site of LCAT and its catalytic mechanism. Since the symposium, the Tesmer laboratory has used electron microscopy to visualize LCAT bound to the edge of discoidal HDL particles (12).

## PATIENTS WITH FLD

### Finland (H. E. Miettinen)

Three families with FLD have been identified in Finland (13–15). The common feature of the index patients was low serum HDL-cholesterol concentration. Otherwise their phenotypes and serum and lipoprotein lipid compositions differed greatly.

In family 1, corneal opacities, anemia, and stomatocytes (abnormal erythrocytes) in peripheral blood were present. One of the subjects had mild proteinuria. Affected family members were compound heterozygotes for LCAT R399C and exon one C-insertion mutations. In family 2, the proband had anemia and an increased number of stomatocytes in his peripheral blood. There were no other clinical findings. He was homozygous for the LCAT R399C mutation, which underlines a phenotype with characteristics of both FLD and FED. In family 3, the proband had corneal opacifications, mild microalbuminuria, and anemia with target cells in his peripheral blood. He was homozygous for the LCAT G230R mutation, designated as LCAT Fin, which represents a complete deficiency (FLD).

Frequencies of these LCAT mutations were examined in Finnish subjects with extremely low serum HDL-cholesterol concentration (<0.7 mmol/l, n = 156). The LCAT Fin mutation was found in 5% of the subjects with low HDL-cholesterol, and it represents the most common genetic cause of reduced HDL-cholesterol levels in southwestern Finland (6). There was no evidence suggesting association of LCAT mutations with coronary artery disease.

### Brazil (B. E. P. Balbo and C. A. T. L. Sampaio)

Based on the identification of three unrelated cases of FLD from the same geographical region in Northern Brazil, Balbo and Sampaio developed an active search program to identify at-risk individuals by clinical and genetic screening. This retrospective study included clinical, laboratory, and molecular genetic analyses of 38 FLD patients from seven different families. Twenty males and 18 females had the diagnosis of FLD established at a mean age of 38.6 ± 16.4 years. Three pathogenic mutations in the LCAT gene were identified, each of them corresponding to a distinct geographic disease cluster: c.G803A (p.R244H), c.C4920T (p.T274I), and c.A2822T (p.I203F).

Phenotypic analysis was performed in all FLD patients. HDL-cholesterol was ≤0.26 mmol/l in all patients, while the mean apoA-I and apoB levels were 0.45 ± 0.10 g/l and 0.41 ± 0.19 g/l, respectively. LDL-cholesterol was >3.75 mmol/l in two patients and triglycerides <1.7 mmol/l in 15 patients. Corneal opacities were detected in 36 cases. Scheimpflug densitometry was performed in three individuals, revealing high corneal density. Anemia was observed in 25 cases (Hb of 10.7 ± 2.2 g/dl) and moderate to severe anemia was detected in almost all patients with advanced renal insufficiency. Hemolysis, detected by indirect bilirubin levels, was observed in 20 individuals. LDH and haptoglobin levels, however, were abnormally elevated and reduced, respectively, in only two patients. Decreased resistance of red blood cells to osmotic stress was found in all five evaluated patients; in their samples, the percentage of hemolysis in progressively lower concentrations of sodium chloride was below the lower reference range for the standardized test, indicating osmotic fragility in the red blood cells. A high intra- and interfamilial variability in estimated glomerular filtration rate was detected and seven patients developed end-stage kidney disease at a median age of 38.3 ± 14.2 years. Systemic arterial hypertension was diagnosed in 22 cases, being associated with age >30 years and estimated glomerular filtration rate <83 ml/min/1.73 m^2^ (CKD-EPI).

Of the 38 patients, eight patients underwent kidney biopsy, which revealed glomerular deposits in all cases and glomerular sclerosis in seven cases. Endocapillary proliferation and glomerular basement membrane splitting were present in four cases, while interstitial fibrosis varied from <5% to 60%. The biopsies revealed thrombotic microangiopathy in three cases and dominance/codominance of C3 mesangial deposits in all analyzed samples. In addition to the effect of abnormal cholesterol deposition in the kidney, these data suggest that thrombotic microangiopathy and C3 deposits may also play a role in the FLD-associated renal injury, supporting a pathogenic effect related to the activation of the alternative complement pathway.

### Norway (E. H. Strøm)

There are four patients identified in Norway. Here, we present the case of a 22-year-old male patient with edema, hypertension, hematuria, and nephrotic proteinuria. A renal biopsy revealed changes consistent with LCAT deficiency. The patient’s lipid profile was abnormal, with especially low HDL-cholesterol 0.3 mmol/l (normal range 0.8–2.1 mmol/l). He was compound heterozygous for mutations R244H and M252K in exon 6 of the LCAT gene and given a diagnosis of FLD. His mother was heterozygous for the M252K mutation, while the father was heterozygous for the R244H mutation.

At the age of 28, he received a kidney graft from his 58-year-old father. Two days after transplantation, he had a biopsy-proven rejection. Electron microscopy of this biopsy showed manifestations of LCAT deficiency. Protocol biopsies taken at 6 weeks and 1 year after transplantation demonstrated progression of deposits. Renal function remained stable. The renal morphology of this disease is characteristic, and the diagnosis should be suspected from the ultrastructural findings (16).

## A WORLDWIDE REGISTRY ABOUT ALL THE KNOWN PATIENTS WITH FLD (I. Wiig)

As of today, less than 100 patients are known. The Norwegian National Advisory Unit on Rare Disorders was asked to take initiative to establish a worldwide contact registry on FLD. This proved to be difficult. Registries on diseases are subject to strict national and international regulations like the European General Data Protection Regulation (17). As a consequence, contact lists for patients have to be organized by patient organizations or through social media groups by patients themselves. The Norwegian National Advisory Unit on Rare Disorders has developed a national quality registry on rare congenital or genetic diseases. Entering consenting patients to the registry is done by medical staff and started in 2019. LCAT deficiency is also incorporated in a workgroup for metabolic nephropathies in the European Reference Network for Rare Kidney Diseases, ERKNet.

## GENETIC FLD AND ATHEROSCLEROSIS (J. A. Kuivenhoven and L. Calabresi)

Based on epidemiological findings, the severe deficiency of HDL in LCAT-deficient carriers should dramatically increase their risk of developing CHD; however, imaging studies evaluating carotid intima-media thickness (IMT), a validated surrogate marker for atherosclerotic CHD, have not provided such evidence. The first study on LCAT-deficient cases from a large Canadian kindred followed for more than 20 years, revealed a slightly increased carotid IMT, which did not progress over 4 years (18). In a subsequent study of five Dutch FED families, the inheritance of LCAT gene mutations only caused a modest increase in carotid IMT (19). The Dutch heterozygotes also showed accelerated carotid atherosclerosis when assessed by 3.0 T magnetic resonance imaging and increased arterial stiffness (20, 21). In contrast, a study of Italian FLD families showed that the inheritance of a mutated LCAT genotype had a remarkable gene-dose-dependent effect in reducing carotid IMT (22), whereas a subgroup of these carriers also showed normal flow-mediated dilation (23). Despite the obvious difficulties in determining the prevalence of CHD in individuals with rare genetic disorders, and the fact that carotid IMT remains a surrogate marker of clinical CHD, the carotid IMT studies in carriers of LCAT gene mutations suggest that FLD does not necessarily accelerate atherosclerosis. In other words, a complete absence of LCAT activity and related near-complete HDL deficiency appears to have no or even a positive impact on atherogenesis (24).

## NON-CLASSICAL METABOLIC PHENOTYPES OF FLD: LESSONS FROM MURINE MODELS (D. Ng)

Loss-of-function mutations causing FLD in humans are inherited in an autosomal codominant fashion. FLD is characterized by the near absence of LCAT activity, HDL-cholesterol, and plasma apoA-I, as well as an elevated unesterified/total cholesterol ratio. Corneal opacity and anemia are highly prevalent, whereas progressive chronic glomerulopathy has been reported in some affected subjects. Studies on genetically engineered LCAT-null mice in the Ng laboratory and others have recapitulated the majority of the aforementioned characteristics. Moreover, the severity was accentuated when crossed into specific transgenic models of dyslipidemia. During the course of studying these model animals, they unexpectedly observed protective phenotypes from diet-induced obesity, insulin resistance, and nonalcoholic fatty liver disease, attributable to loss of LCAT (25). Further analyses uncovered an important role of cellular cholesterol in modulating the pathogenesis of nutritional excess-induced ER stress, insulin resistance, steatohepatitis, and ectopic brown adipose tissue biogenesis. Specifically, they learned that hepatic ER stress is strongly correlated with cholesterol content in the hepatocyte ER but not in other cell compartments (26). On the other hand, cholesterol from endogenous synthesis and dietary sources both contribute to hepatic inflammasome activation (27), in part through cholesterol crystal formation. The findings also uncovered a critical role for cellular cholesterol in modulating the spontaneous development of classical brown adipose tissue in skeletal muscle from myoblasts in utero and satellite cells in adults (28).

## LCAT THERAPEUTIC INNOVATIONS (M. Zhou)

Innovations in LCAT therapeutics led to the identification of three modalities for increasing LCAT activity: a small molecule activator (29), a modified recombinant LCAT protein (30), and an agonistic human monoclonal antibody (31). Lead molecules from these three distinct modalities all robustly increase LCAT enzymatic activity and modulate HDL metabolism, while each presents unique properties to meet different clinical needs. Studies aiming at understanding the mechanism of LCAT activation identified a cysteine residue that plays a crucial role in regulating LCAT catalytic activity. Incremental hypothesis testing in the proof-of-concept studies showed that LCAT activation modulates HDL particle composition and size, promotes reverse cholesterol transport, and attenuates the progression of atherosclerosis in preclinical disease models.

## TREATMENT OF FLD PATIENTS WITH RECOMBINANT LCAT (A. T. Remaley)

Because of the success in using enzyme replacement therapy for the treatment of several other rare genetic disorders, there has been a recent effort to also develop recombinant human LCAT (rhLCAT) as a therapy. The two main potential indications for rhLCAT would be for the treatment of FLD and for raising HDL-cholesterol for the possible treatment of cardiovascular disease.

Although FLD patients can develop severe anemia and corneal opacities, the main cause of morbidity and mortality is renal disease. In animal models, it has been shown that LpX, which only forms with the near complete absence of LCAT, is nephrotoxic and promotes the development of renal disease (4). After intravenous infusion, LpX gets trapped in the glomerulus where it damages podocytes and causes proteinuria in LCAT-KO but not wild-type mice. Furthermore, in vitro incubation of LpX with rhLCAT causes its dissolution and accelerates its catabolism in mice (32). In addition, a single intravenous treatment of LCAT-KO mice with rhLCAT markedly raises HDL-cholesterol and normalizes their lipoprotein profile for several days (33).

Besides its possible utility for the treatment of FLD, rhLCAT may also be beneficial for cardiovascular disease because of its role in reverse cholesterol transport. Based on this concept, and many promising animal studies showing that HDL infusion can rapidly reverse atherosclerotic plaques, there have been several clinical trials involving the infusion of reconstituted preβ-HDL-like particles (rHDLs) in patients with acute coronary syndrome (34). However, the lipid composition used in manufacturing such rHDL therapies appears to significantly affect their functionality and ultimate efficacy in the clinic (35). In transgenic rabbits that express human LCAT (36) or in rabbits treated with rhLCAT, it has been shown that LCAT can protect against diet-induced atherosclerosis. It should be also noted that, in contrast to rabbits that naturally express CETP, overexpression of LCAT in mice, which do not naturally express CETP, promotes atherosclerosis, emphasizing the importance of CETP in cholesterol processing following the LCAT reaction (37).

Based on the above rationale, rhLCAT produced in CHO cells has been developed and tested in two human studies (sponsored by AlphaCore Pharma). Plasma LCAT concentration rapidly rose after the infusion and was dose-proportional and had an estimated terminal half-life of 42 h. The low dose of rhLCAT did not change HDL-cholesterol levels, but HDL-cholesterol increases were observed with the other doses and increased as much as 50% in some patients on the highest dose. Preβ-HDL sharply dropped after the infusion, consistent with conversion to larger HDL particles due to cholesterol esterification, which was confirmed by electrophoresis and by NMR. The rhLCAT treatment also increased the capacity of LDL-depleted serum to promote cholesterol efflux from J744 macrophage cells. Most of the plasma lipid changes returned to baseline after approximately 3–4 days. The overall conclusion from this study was that rhLCAT has an acceptable safety profile and the expected effect on HDL parameters and thus supported its future development.

The other human study on rhLCAT was a single patient study of an FLD patient with advanced renal disease and nearly undetectable HDL-cholesterol levels (38). The patient was treated every week to every 2 weeks over an approximately 7 month period, with a dose ranging between 3 and 9 mg/kg. Treatment with rhLCAT nearly normalized HDL-cholesterol levels and it returned to baseline after about 3 days. In contrast, the percent of cholesteryl esters in plasma, which also normalized after the treatment, remained elevated for over 1 week. In terms of renal function, the patient showed some minor improvements within the first month of treatment but did not show any further improvement thereafter. Before starting treatment, the patient had severe anemia, but it significantly improved from 8.2 to 10.1 g/dl after a few months of rhLCAT treatment. It is difficult to conclude much from this single patient study, but it suggests that unlike renal function, the lipid abnormalities and anemia in FLD may quickly respond to rhLCAT treatment. After the onset of proteinuria, it typically takes several decades for FLD patients to develop end-stage renal disease, so the early identification of this disorder and early treatment with rhLCAT could hopefully prevent this outcome.

## ACUTE LCAT INFUSION INCREASES MACROPHAGE-SPECIFIC CHOLESTEROL EFFLUX IN LCAT-DEFICIENT HUMAN apoA-I TRANSGENIC MICE (S. K. Karathanasis)

To determine if a single IV injection of rhLCAT induces whole body macrophage-specific cholesterol efflux, LCAT-KO/human apoA-I transgenic mice (LCAT-KO×hapoA-I Tg) were treated with vehicle or rhLCAT followed 10 min later by injection with 3H-cholesterol/BSA nanoparticles. Plasma was collected via retro-orbital bleeding at 1, 4, 24, and 48 h after injection. At the end of the study, all animals were euthanized and organs (liver and spleen) were collected. rhLCAT treatment significantly increased (>3-fold) the rate of 3H-cholesterol tracer reappearance in the plasma, while it decreased tracer appearance in the liver and spleen. Also, rhLCAT treatment significantly increased plasma HDL-cholesterol mass that was mostly cholesteryl ester, suggesting the formation of large mature HDL particles. rhLCAT treatment did not affect plasma LDL-cholesterol levels or fecal tracer excretion. The lack of endogenous CETP in mice may have limited the effective delivery of cholesterol to LDL and its eventual uptake and excretion by the liver. These results, for the first time, show that recombinant LCAT infusion stimulates cholesterol efflux in vivo and suggest that it could also enhance cholesterol efflux from macrophages in atherosclerotic plaques. Several diseases are associated with reduced LCAT mass and/or activity, including genetic LCAT deficiencies, ischemic heart disease, chronic kidney disease, and certain liver diseases. Our current data suggest that rhLCAT treatment may be of therapeutic value in these diseases.

## PERSPECTIVES

Although FLD is a very rare disease, the fact that 38 patients from seven different families in Brazil were recently identified suggests that the disease is underdiagnosed and may be more prevalent in isolated geographical regions. Regardless, studying its underlying pathophysiology has revealed important insights in lipoprotein metabolism and function, particularly regarding LCAT, in the context of a variety of diseases beyond FLD. For example, it was recently reported that rhLCAT rescues the defective HDL functionality to promote endothelial NO production in patients with acute coronary syndrome (39). Such findings support clinical evaluation of rhLCAT in patients with myocardial infarction and other cardiovascular diseases involving endothelial cell dysfunction. Similarly, recently published data using an animal model of chemically induced primary biliary cirrhosis suggests that rhLCAT treatment may be effective in reducing plasma LpX levels and reduce the risk for xanthoma formation, plasma hyperviscosity, and, perhaps, neuropathies in patients with primary biliary cirrhosis (40). Although more basic work is required to understand whether these beneficial effects of LCAT are exclusively due to its effects in improving HDL function or additional direct enzymatic effects in diseased tissues, rhLCAT therapy appears to be promising and the ongoing clinical studies may reveal new insights to guide patient selection and drug optimization and use.

## CONCLUDING REMARKS

Fifty years after the discovery of the first patients with FLD, the first international meeting was held in May 2017 on LCAT. This meeting brought together researchers and clinicians interested in the role of LCAT in both healthy patients and those with FED and FLD. LCAT catalyzes the conversion of free cholesterol to cholesteryl esters on HDL particles, generating a cholesterol concentration gradient that promotes cholesterol outflow from cell membranes. This concentration gradient may enhance cholesterol efflux from cells and thus may prevent the development of atherosclerosis. Structural studies of LCAT and, specifically, recent electron microscopy studies probing the LCAT-HDL complex are guiding the understanding of LCAT activation by apolipoproteins as well as aiding in the development of future therapeutics. rhLCAT produced in CHO cells has been developed and tested in two human studies with promising improvements in both correcting the lipoprotein profile and anemia. Any effect on renal function was, however, less convincing and suggests that treatment before the onset of renal disease may be critical. Patients with FLD offer an important opportunity for the understanding of cholesterol metabolism because LCAT is essential for the normal metabolism of cholesterol in lipoproteins. The role of LCAT in atherosclerosis is not yet fully understood, underpinning the importance of studying patients with FLD in more detail. As suggested throughout this report, increasing knowledge of the role of LCAT in human health is also expected to allow for the development of a better treatment for FLD.
